# Improving the antinutritional profiles of common beans (*Phaseolus vulgaris* L.) moderately impacts carotenoid bioaccessibility but not mineral solubility

**DOI:** 10.1038/s41598-024-61475-8

**Published:** 2024-05-24

**Authors:** Katherine Alvarado-Ramos, Ángela Bravo-Nunez, Charlotte Halimi, Matthieu Maillot, Christèle Icard-Vernière, Chiara Forti, Chiara Preite, Luisa Ferrari, Tea Sala, Alessia Losa, Eleonora Cominelli, Francesca Sparvoli, Emanuela Camilli, Silvia Lisciani, Stefania Marconi, Stephane Georgé, Claire Mouquet-Rivier, Karl Kunert, Emmanuelle Reboul

**Affiliations:** 1 Aix-Marseille Université, INRAE, INSERM, C2VN, Marseille, France; 2 MS-Nutrition, Marseille, France; 3 Institute of Agricultural Biology and Biotechnology, National Research Council, Milan, Italy; 4 QualiSud, Université de Montpellier, Université d’Avignon, CIRAD, Institut Agro, IRD, Université de la Réunion, Montpellier, France; 5 Centre Technique de Conservation des Produits Agricoles, Avignon, France; 6 Council for Research in Agriculture and Economics, Research Centre for Genomics and Bioinformatics, Montanaso Lombardo, Italy; 7 Council for Agricultural Research and Economics, Research Centre for Food and Nutrition, Rome, Italy; 8 Department of Plant and Soil Sciences, Forestry and Agricultural Biotechnology Institute, University of Pretoria, Pretoria, South Africa; 9 University of Valladolid, Valladolid, Spain

**Keywords:** Fat metabolism, Iron

## Abstract

Common beans are a common staple food with valuable nutritional qualities, but their high contents in antinutritional factors (ANFs) can decrease the bioavailability of (i) fat-soluble micronutrients including carotenoids and (ii) minerals. Our objective was to select ANF-poor bean lines that would not interfere with carotenoid and mineral bioavailability. To achieve this objective, seeds of commercial and experimental *Phaseolus vulgaris* L. bean lines were produced for 2 years and the bean’s content in ANFs (saponins, phytates, tannins, total polyphenols) was assessed. We then measured carotenoid bioaccessibility and mineral solubility (i.e. the fraction of carotenoid and mineral that transfer into the aqueous phase of the digesta and is therefore absorbable) from prepared beans using in vitro digestion. All beans contained at least 200 mg/100 g of saponins and 2.44 mg/100 g tannins. The low phytic acid (*lpa*) lines, *lpa1* and lpa1^2^ exhibited lower phytate levels (≈ − 80%, *p* = 0.007 and *p* = 0.02) than their control BAT-93. However, this decrease had no significant impact on mineral solubility. HP5/1 (*lpa* + phaseolin and lectin PHA-E free) bean line, induced an improvement in carotenoid bioaccessibility (i.e., + 38%, *p* = 0.02, and + 32%, *p* = 0.005, for phytofluene bioaccessibility in 2021 and 2022, respectively). We conclude that decrease in the phytate bean content should thus likely be associated to decreases in other ANFs such as tannins or polyphenols to lead to significant improvement of micronutrient bioaccessibility.

## Introduction

The world's population is estimated to increase to 9.6 billion in 2050 in a context of climate change^1^. This alarming observation has pushed both scientists and industry to find sustainable solutions to improve nutrition. Increasing the proportion of pulses in our diet is widely recognized as a key factor in designing healthy sustainable diets for the future.

*Phaseolus vulgaris* L., or common bean, is a pulse commonly used as a staple food in seed form throughout the world. Due to the bean’s high protein content, common bean consumption can participate in reducing both meat consumption and cost of nutrition^2^. Common beans are rich in some B vitamins and minerals, but also contain bioactive compounds that can limit the assimilation of proteins, minerals, and vitamins. Due to these properties, these compounds are sometimes called antinutritional factors (ANFs)^3^. These ANFs include (i) phytates that can bind divalent cations (iron, magnesium, zinc) and proteins, limiting their digestion^4^, (ii) polyphenols and tannins that can also bind both cations and proteins^5^, (iii) saponins that can associate to lipid compounds such as cholesterol or to minerals and in turn reduce their intestinal absorption^6^, and (iv) the abundant seed storage protein lectin phytohemagglutinin PHA, which consists of 2 subunits (PHA-E and PHA-L, respectively), and which is known to interfere with iron absorption in animal studies^7^.

The presence of these ANFs in beans can thus decrease both mineral, fat-soluble vitamin and carotenoid bioavailability present in the same meal, as previously confirmed in vitro^8,9^.

Different treatments eliminate ANFs or reduce their activity (heat treatment, high pressure, hydration, fermentation) but these methods do not eliminate/inactivate the ANFs completely^3,10,11^. Decreasing bean ANF content by selecting optimized bean lines can be an interesting option to increase the bean’s nutritional properties. Beans with low phytic acid, i.e. *lpa* mutants^12,13^, or beans without phaseolin/PHA-E have previously been developed^14^. In this study, we used three groups of bean lines:

- ***lpa* mutants** The *lpa1* mutation^15,16^ was introduced in the BAT 93 background (CIAT, Cali, Colombia), as described by^17^. The *lpa1*^2^, *lpa2*, *lpa3* and *lpa4* mutants were isolated as lines with a high inorganic phosphate content through a screening of an EMS mutagenized common bean collection, as described previously^17^. The *lpa1*^2^ mutant was characterized as an *lpa* mutant allelic to *lpa1* and described in details elsewhere^17^.
- **Phaseolin and/or lectin and/or *lpa* mutants** Phaseolin, the most abundant protein in beans, is not considered an ANF. However, its low digestibility has encouraged researchers to reduce its content in bean lines to improve their nutritional quality^14^. In this group, BAT 881 hybrid, created by CIAT, Colombia^18–20^ was considered as a control as it contains phaseolin, PHA-L and PHA-E. Line 938 was obtained as previously described^15^ and contains phaseolin and PHA-L, but neither PHA-E nor α-amylase inhibitor (a lectin-like protein). Finally, HP5/1 is also a *lpa* mutant harboring the same mutation as *lpa1* but in another genetic background^14^. This line contains PHA-L but neither phaseolin nor PHA-E and α-amylase inhibitor.
- **Commercial lines** Meccano and Mercato bean varieties were purchased from Blumen (Piacenza, Italy) and selected because of their capacity to resist high temperatures^21,22^. PAN-123 and PAN-146 varieties were purchased from Pannar (South Africa).

The different bean lots were prepared in a recipe containing canned tomato pulp and red bell peppers. This recipe was chosen to mimic a meal rich in carotenoids. Our objective was to determine whether changes in bean ANF composition could lead to an improvement of the bioaccessibility/solubility of carotenoids and minerals ingested within the same meal using in vitro digestion.

## Material and methods

### Chemicals

Retinyl acetate, β-carotene, α-carotene, vitamin D_3_ (> 95% pure), deuterated vitamin D_3_, 4-phenyl-1,2,4-triazoline-3,5-dione (PTAD), phytic acid, DMACA, vanillin, iron III, pancreatin, ammonium thiocyanate, catechins, α-amylase, pepsin, bile, ferric chloride and sodium carbonate were purchased from Sigma Aldrich (Saint-Quentin-Fallavier, France). Lutein, lycopene, phytoene and phytofluene were purchased from Extrasynthèse (Genay, France). Soyasaponin I was generously supplied by Stéphane Georgé, CTCPA Avignon. Canned tomato pulp, red bell peppers and Isio4 oil (Lesieur, Asnières, France) were purchased from a local supermarket (Marseille, France). Methanol, acetonitrile, hexane, dichloromethane, methyl-tert-butylether, acetone, ethanol absolute anhydrous, sulfuric acid, hydrochloric acid, nitric acid, formic acid, hydrogen peroxide were purchased from CarloErba Reagents (Peypin, France).

### Bean production

The experimental and commercial bean seeds were germinated in greenhouses, then the 3–4-week-old seedlings were grown in open fields in Montanaso Lombardo (45°20′12″N 9°28′11″E, CREA GB, Italy), during Spring–Summer 2021 and 2022. Eight bean lines were produced in both 2021 and 2022 (BAT 93, *lpa1*^2^, *lpa4*, BAT-881, HP5/1, Meccano, Mercato). Five additional bean lines were produced in 2022 (*lpa1*, *lpa2*, *lpa3*, PAN-126 and PAN-143). After harvest, the seeds were placed at – 20 °C for 3 weeks to prevent the action of *Acanthoscelides obtectus* (weevil), which spoils the seeds. Finally, the seeds were dried in an oven at 37 °C for five days.

### Bean cooking

A first set of experiment was performed to determine bean cooking time. Briefly, samples of 20 seeds were soaked for 16 h in distilled water at room temperature. A Mattson pin drop cooker (Michigan State University Department of Physics and Astronomy Machine Shop) was then used to measure cooking times of soaked seeds^23^. The cooker containing 25 seeds (20 seeds from the same sample and 5 control seeds) from a single sample was placed in a glass beaker with boiling distilled water set on a hot plate. Individual beans were considered cooked when the piercing rod had passed through a seed. The sample cooking time was recorded when 80% of the pins pierced the beans. Two technical replicates were evaluated for each sample.

Bean cooking was then performed in an experimental kitchen (CREA AN, Italy). To this aim, approximately 300 g of dried beans were soaked for 17.5 h at 20 °C in 1 L of distilled water. After draining soaking water, beans were cooked in a pot with approximately 2 L of boiling distilled water, over moderate heat, to mimic home cooking. Each sample was cooked until beans felt tender when pierced with a fork, considering the reference cooking times previously established. Remaining cooking water was drained. Beans were then vacuum-sealed in plastic bags and were used withing 3 weeks.

### Lyophilization and ANFs quantification

Once the vacuum-bags of beans were opened for in vitro digestion, a fraction of beans was lyophilized for 7 days in a Christ-alpha manifold 100400 freeze-dryer from Fischer Scientific. Then, beans were dry milled in a Pulverisette 2 (Fritsch, Idar-Oberstein, Germany) and stored in plastic tubes at room temperature until antinutrient analysis (saponins, phytates, tannins, and total polyphenols).

#### Saponins

Triterpene saponins were analyzed using a spectrophotometric method adapted from^24^. Briefly, 1 g of sample was weighed separately and mixed with 5 mL of 80% methanol diluted in ultrapure water at room temperature for 24 h. The mixture was then centrifuged for 10 min at 10 °C and 4500 rpm. The supernatant was recovered and placed in new tubes. The procedure was repeated twice, and the supernatant was recovered in the same tube and homogenized. The supernatant (0.125 mL) was mixed with 0.125 mL of 80% methanol and 0.250 mL of 8% vanillin diluted in pure ethanol absolute anhydrous. Tubes were placed in an ice bath, and 2.5 mL of 72% sulfuric acid diluted in water was added. After 15 min, the tubes were incubated for 10 min at 60 °C in a shaking incubator. Then the tubes were placed back in the ice bath. A calibration curve was obtained using a soyasaponin I standard, diluted in 80% methanol and treated in the same way as the samples. The absorbance was measured at 544 nm in a spectrophotometer (Shimadzu UV-1800) against a solution containing the same amounts of methanol, vanillin, and sulfuric acid as the samples. All samples were measured by triplicate.

#### Phytates

Phytate quantification was performed using a spectrophotometric method adapted from^25^. Briefly, 0.2 g of sample was mixed with 10 mL of 0.5 M hydrochloric acid (to achieve a high extraction percentage) at room temperature for 60 min. The tubes were then centrifuged for 10 min at 4 °C and 4500 rpm. The supernatant was extracted to another tube and refrigerated at 4 °C until measurement on the same day. The complex of iron(III) thiocyanate was prepared using: 25 mL of ferric chloride (300 μg/mL), 25 mL of ammonium thiocyanate (150 mg/mL), 0.2 mL of concentrated nitric acid, and water to complete 100 mL. The calibration curve was prepared using a solution of phytic acid 1 g/L, diluted with ultrapure water and 0.5 M hydrochloric acid (the same volume of the dilution to obtain a condition similar to the samples). A blank tube was prepared with 100 μL of 0.5 M hydrochloric acid and 900 μL of ultrapure water for the spectrophotometer method. 2 mL of the iron(III) thiocyanate complex was added to 0.1 mL of the supernatants, 0.9 mL of ultrapure water in tubes, the blank tube, and 1 mL of the calibration curve tubes. All tubes were stirred at 40 °C for 2 h and 30 min, then cooled to room temperature and centrifuged for 5 min at 4 °C and 4500 rpm. The absorbance was measured against the blank at 480 nm in a spectrophotometer (Shimadzu UV-1800). All samples were measured in triplicate.

#### Tannins

The tannin assay was adapted from^26^ and^10^. Briefly, 0.12 g of samples were extracted in 12 mL of ultrapure water, with agitation for 30 min. After agitation, the samples were centrifuged for 10 min at 4 °C and 4500 rpm. The supernatants were transferred to new tubes. The calibration curve was prepared from a catechin solution at a concentration of 500 mg/L, diluted in pure methanol and water 1:1 (v/v). DMACA was prepared the day of the experiment from a solution of 2% (w/v) DMACA in pure methanol and 6 M hydrochloric acid 1:1 (v/v). DMACA solution (9 mL) were required and then diluted in pure methanol to a total volume of 25 mL. In new tubes, 4 mL of the sample extract (homogenized), calibration, and reference solutions were added individually, followed by 1 mL of DMACA solution and 2 mL of 50% methanol diluted in water 1:1 (v/v) to all tubes. All tubes were mixed for 20 min at room temperature. The absorbance was measured at 640 nm. All samples were measured in triplicate.

#### Total polyphenols

Total polyphenols were measured using the Folin-Ciocalteu method, optimized and adapted to plant-derived products^27^. Samples (1 g) were extracted with a 7:3 (v/v) solution of acetone and water for 30 min. The supernatants were filtered and placed into new tubes (raw extracts). The acetone content of the mixture was reduced to 7% using distilled water, then 2 mL of the mixture were placed on an Oasis cartridge and filtered (washing extracts). A fraction of the washing extracts was then incubated for 2 h at 85 °C in an oil bath (heated washing extracts).

The raw, washing and heated washing extracts were individually added to 2.5 mL of Folin-Ciocalteu reagent diluted 1:10 (v/v) in water and incubated for 2 min at room temperature. Then 2 mL of sodium carbonate (75 g/L) was added. All tubes were incubated for 15 min at 50 °C. After incubation, the tubes were cooled in an ice bath and the absorbance was measured briefly at 750 nm by HPLC (gradient of 0.5% water, formic acid, and acetonitrile). The results were finally expressed in gallic acid equivalents.

### In vitro digestion

Fresh red bell peppers were first cooked in a pan for 45 min with a spoon of water. Beans were then added to canned tomato pulp and cooked red bell peppers. Isio4 oil was supplemented with vitamin D_3_ at a concentration of 0.14 mg/100 mg of oil and was added just before the beginning of the digestion assay. Each test meal contained 3 g of cooked beans, 1.5 g of cooked red bell peppers, 1.5 g of canned tomato pulp and 100 µL of oil. In vitro digestions were conducted by mimicking an oral, a gastric and a duodenal step as previously described^28^, except that all volumes were divided by two. The digesta obtained at the end of the duodenal phase was centrifuged at 2500 rpm for 1 h at 10 °C to isolate the aqueous phase of the digesta, which contained the absorbable compounds (fat-soluble compounds included in mixed micelles and soluble minerals). Aliquots of the digestas and aqueous phases were frozen at – 80 °C until analysis. In vitro digestions were performed in quadruplicate for each sample.

### Carotenoid extraction and quantification by HLPC

A double hexane extraction was performed to extract carotenoids from each sample (digesta or aqueous phase). Briefly, 500 μL aliquots of the samples were mixed with 500 μL of retinyl acetate in ethanol (internal standard). Hexane (2 mL) were added and the mixture was vortexed for 10 min. Then, all samples were centrifuged for 10 min at 2500 rpm, 4 °C. The hexane phase was carefully removed and transferred to another tube. The process was repeated twice and the second extracted phase was pooled with the first one. Once dried, the residue was recovered with 200 μL of methanol and dichloromethane (65/35, v/v). The mixture was vortexed and transferred to a vial for HPLC analysis. The high-performance liquid chromatography (HPLC) analysis was carried out on a Thermo Scientific UltiMate 3000 with a C30 column at 40 °C according to a previously published protocol^29^. A volume of 50–80 μL was injected. Identification of carotenoids was performed with Chromeleon 7.2 (ThermoFischer Scientific). Quantification was done by comparing the peak areas with standard curves.

Carotenoid bioaccessibility was defined as the percentage of the amount of carotenoids found in the aqueous phase divided by the amount of carotenoids found in the digesta.

### Vitamin D extraction and quantification by LC-MS^2^

Samples were stored at – 80 °C before analysis. The protocol was adapted from^30^ and 500 μL of sample was required. A solution of deuterated cholecalciferol in ethanol (20 μL) at a concentration of 0.4 ng/μL was also required. The extracted cholecalciferol was derivatized with a solution of PTAD in acetonitrile at a concentration of 4 mg/mL. PTAD (50 μL) were added to the dry extract, mixed and incubated for 10 min. This step was repeated and stopped at 5 min when 20 μL of ultrapure water was added, mixed and incubated again for 5 min. The mixture was evaporated under nitrogen. The dry residue was diluted in 100 μL of acetonitrile and transferred to a vial for analysis by liquid chromatography coupled to tandem mass spectrometry (LC–MS^2^) using a Thermo Fischer Scientific instrument (Illkirch, France). Chromeleon 7.2 ThermoFischer Scientific software was applied to set up, directly control, and process data. Cholecalciferol was quantified as the ratio of the peak area and the retention time to the result of the deuterated solution.

Vitamin D bioaccessibility was defined as the percentage of the amount of vitamin D found in the aqueous phase divided by the amount of vitamin D found in the digesta.

### Mineral extraction and quantification by ICP-OES

Iron, zinc, magnesium, and copper were extracted from individual ingredients (canned tomato pulp, red bell peppers and beans), digestas, and aqueous phases. Briefly, 0.45 g of solid samples, or 1 mL of digestas or aqueous phases (0.1 g of dry matter), were placed in adequate teflon containers. A solution of 69.5% nitric acid and 30% hydrogen peroxide was added in the following proportions: 7:1 for solid samples and 1.75:0.25 for micro-extractions. After 30 min, the containers were closed and the mixtures were microwave-digested/mineralized using the ETHOS™ EASY Milestone at 1200 Watts for 1 h 15 min (including cooling time). The contents were transferred to plastic tubes, and ultrapure water was added to reach a final volume of 25 mL for solid samples and 6 mL for digestas/aqueous phases, respectively. The final solutions were analyzed with the Agilent Technologies 5100 inductively coupled plasma optical emission spectrometry (ICP-OES) system. All replicates of digestas and aqueous phases were measured once and were calculated as the mean ± SEM of a sample. Ingredients were measured once. ICP-OES carried out three measurements before averaging the results.

Solubility of minerals was calculated as the percentage of the amount of minerals found in the aqueous phase of the digesta divided by the amount of minerals in the digesta.

### Zinc bioavailability estimation

For comparison with the zinc solubility in the digesta, predicted zinc absorption was calculated according to^31^.

For this, we used:

$$TAZ=0.5 \times \left\{0.13+TDZ\right.+0.10\left(1+ \frac{TDP}{1.2}\right)$$

$$-\sqrt{(0.13+TDZ+0.10 (1+\frac{TDP}{1.2}){)}^{2}-4\times 0.13\times \left.TDZ\right\}}$$

where TAZ = total absorbed zinc (mmol), TDZ: total dietary zinc (mmol), TDP: total dietary phytates (mmol). For this purpose, we used the zinc content and phytates content measured in our samples. TAZ was estimated for a bean portion of 200 g. Then zinc absorption (ZA) was calculated as the ratio TAZ/TDZ:

$$ZA=\frac{TAZ}{TDZ}$$

### Statical analysis

Carotenoid bioaccessibility and mineral solubility obtained with BAT 93 were set to 100% and all other bioaccessibility and solubility values were expressed relative to BAT 93 condition.

After verifying the result's nature, the Kruskal–Wallis test was applied to test the differences between genotypes in groups of more than 3 samples and a Wilcoxon-Mann Whitney test for groups with only 2 samples. If a significant difference was found, a Nemenyi test was applied as a post hoc test. Results were expressed as means ± SEM. Values were considered significant when p < 0.05 (alpha 5%, IC = 95).

For all comparations, each genotype was tested against its control, and commercial lines were tested against BAT 93. Principal component analysis (PCA) was applied to summarize and visualize the dataset, and then identify correlations between the variables. R Studio version 4.3.2 was used for the analysis.

## Results

### Nutritional profiles of the ingredients for in vitro digestion

Table 1 shows the ANF, carotenoid and mineral contents of both ingredients (canned tomato pulp, red bell peppers) and cooked bean samples.

**Table 1:**
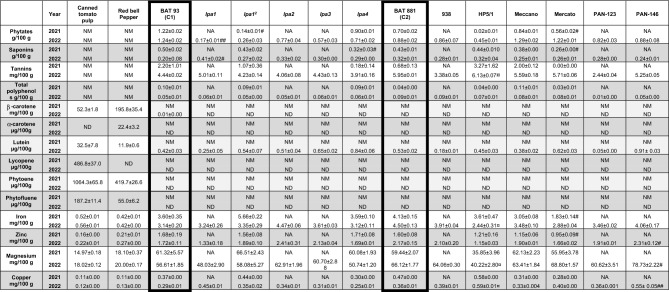
Nutritional and antinutritional profiles of ingredients used for in vitro digestion.

*NA* not available, *ND* not detectable, *NM* not measured. Values are given for cooked red peppers and cooked beans.

BAT 93 (C1) was used as control for *lpa1*, *lpa1*^*2*^, *lpa2*, *lpa3*, *lpa4*. Meccano. Mercato. PAN-123 and PAN-146. BAT 881(C2) as control for 938 and HP5/1. Significant differences against C1 are represented by (#) and for C2 (¤) Significant differences between 2021 and 2022 are represented by (*). Data = mean ± SEM; n = 3 for each group. For all symbols (*, #, ¤): *p < 0.05; **p < 0.01; ***p < 0.001; ****p < 0.0001.

The *lpa* mutant bean lines had less phytates than their control for a given year. *lpa1* and *lpa1*^2^ lines had the lowest phytate amount (≈ − 80% compared to BAT-93, *p* = 0.007 and *p* = 0.02). HP5/1 displayed a lower content in phytates than BAT-881 (> − 50% compared to BAT-881, *p* = 0.09). Finally, in the group of commercial bean lines, Mercato beans contained significantly less phytates and saponins than BAT-93-T beans in 2021 (− 54.1% and − 48.0% for phytates and saponins, respectively, *p* = 0.02, p = 0.02), but these differences were not found in 2022. ANF were not measured in tomato pulp and red bell peppers.

The canned tomato pulp and the red bell peppers used for the in vitro digestion in 2021 and 2022 were from the same lots stored at – 80 °C. Carotenoid analysis showed that canned tomato pulp was rich in lycopene, phytoene and phytofluene (486.8 μg/100 g, 1064.3 μg/100 g, and 187.2 μg/100 g, respectively), and also contained β-carotene and lutein (52.3 μg/100 g and 32.5 μg/100 g, respectively). Red bell peppers contained large amount of β-carotene and phytoene (195.8 μg/100 g and 419.7 μg/100 g, respectively), as well as α-carotene, lutein and phytoene (22.4 μg/100 g, 11.9 μg/100 g, and 55.0 μg/100 g, respectively). Regarding the bean lines, BAT 93 contained a minor amount of β-carotene (0.01 μg/100 g). All the beans contained trace amounts of lutein (from 0.05 to 0.91 μg/100 g).

No significant differences between the *lpa* mutant bean lines and their respective control were found for mineral contents in 2021 and 2022. In the low phaseolin/lectin/phytate line (HP5/1), a lower iron and magnesium content was found when compared to BAT-881 (− 46% and − 39% in 2023, respectively, *p* = 0.02, *p* = 0.03). Mercato beans contained less iron (− 49%,* p* = 0.02) and less zinc (− 44%, *p* = 0.02) than BAT-93 in 2021, but these differences were not observed in 2022. PAN-146, which was only characterized in 2022, had more zinc (+ 92%,* p* = 0.049), magnesium (+ 39%, *p* = 0.01) and copper (+ 90%, *p* = 0.01) than BAT-93.

### Bioaccessibility of carotenoids and vitamin D

**Figure 1:**
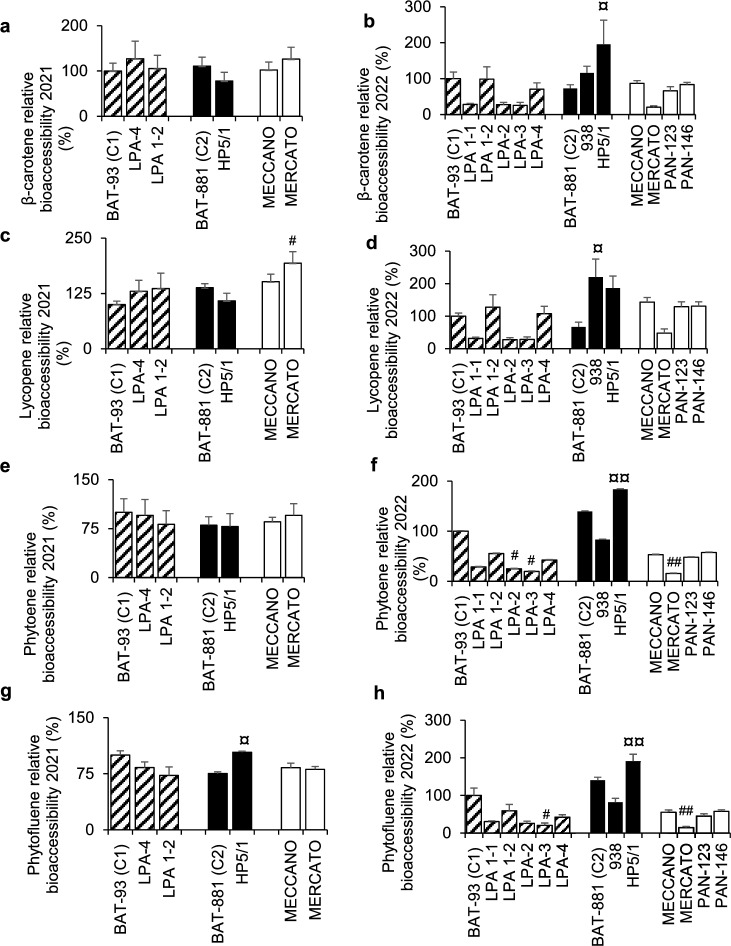
Carotenoid bioaccessibility from test meals containing the different beans lines. Test meals were constituted of cooked beans prepared with oil, tomato pulp and red bell peppers. In vitro digestion was performed to mimic the oral, gastric and duodenal steps. Carotenoid bioaccessibility was defined as the percentage of the amount of carotenoid found in mixed micelles divided by the amount found in the digesta. Data are the mean ± SEM, n = 3 to 4. Significant differences with the control BAT 93 are represented by (#). Significant differences with the control BAT-881 are represented by (¤). For both symbols (#, ¤): *p < 0.05; **p < 0.01; ***p < 0.001; ****p < 0.0001.

In 2021, Mercato beans were associated with a higher bioaccessibility of lycopene when compared to BAT-93 (*p* = 0.02, Fig. 1c). However, this was not found in 2022, where Mercato beans were associated with a lower bioaccessibility of β-carotene (*p* = 0.03, Fig. 1b), phytoene (*p* = 0.002, Fig. 1f), phytofluene (*p* = 0.002, Fig. 1h) compared to its control.

Compared to BAT-881, HP5/1 was associated with a higher bioaccessibility of phytofluene (+ 38%, *p* = 0.02, Fig. 1g) in 2021, and a higher bioaccessibility of β-carotene (+ 169%, *p* = 0.02,

Fig. 1a), phytoene (+ 32%*, p* = 0.005, Fig. 1f) and phytofluene (+ 36%*, p* = 0.005, Fig. 1h) in 2022.

No difference was found regarding vitamin D bioaccessibility (data not shown).

### Solubility of minerals

**Figure 2:**
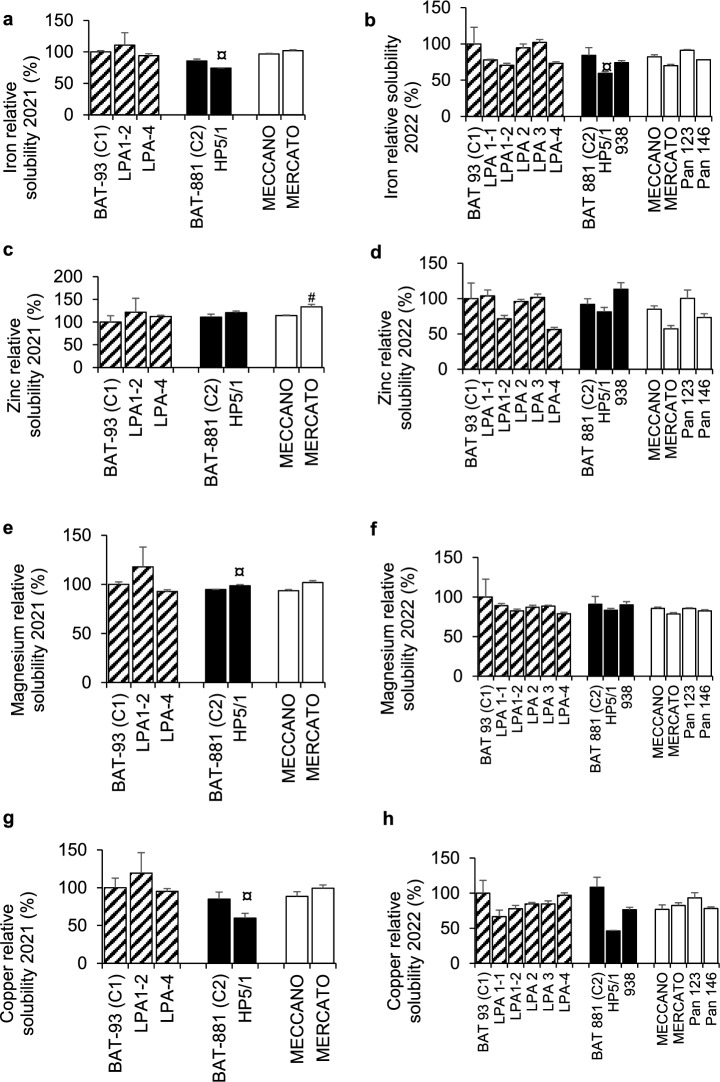
Mineral solubility from test meals containing the different beans lines. Test meals were constituted of cooked beans prepared with oil, tomato pulp and red bell peppers. In vitro digestion was performed to mimic the oral, gastric and duodenal stages. Mineral solubility was defined as the percentage of the amount of mineral found in the aqueous phase divided by the amount found in the digesta. Data are the mean ± SEM, n = 3 to 4. Significant differences with the control BAT-93 are represented by (#). Significant differences with the control BAT-881 are represented by (¤). For both symbols (#, ¤): *p < 0.05; **p < 0.01; ***p < 0.001; ****p < 0.0001.

In 2021, HP5/1 was associated with a lower solubility of iron (− 14%, *p* = 0.02, Fig. 2a) and copper (− 4%, 0.02, Fig. 2g) but a higher solubility of magnesium (+ 29%, *p* = 0.02, Fig. 2e) compared to its control, while test meals containing Mercato beans had a higher zinc solubility (+ 34, *p* = 0.02, Fig. 2c) compared to test meals containing BAT-93. The only effect confirmed in 2022 was the lower iron solubility (− 29%, *p* = 0.049, Fig. 2b) observed for test meals containing HP5/1 compared to its control.

The prediction of total absorbed zinc (TAZ) was significantly higher for *lpa1*^2^ mutants than for its control BAT-93 (+ 50%, *p* = 0.02) in 2021 and 2022. Compared to their control BAT-93, zinc absorption (ZA) was higher in *lpa1*^2^ and Mercato beans (*p* = 0.02,* p* = 0.02) in 2021 and *lpa1* (*p* = 0.008) in 2022 (Table 2).

**Table 2:** Zinc bioavailability estimation of bean lines in 2021 and 2022.

Bean line	Total absorbed zinc (TAZ, 10^–3^ mmol)		Zinc absorption (ZA)	
	2021	2022	2021	2022
*BAT 93 (C1)*	11.6 ± 0.8	11.7 ± 0.5	0.225 ± 0.002	0.222 ± 0.002
* lpa 1*		17.8 ± 1.2		0.438 ± 0.007^##^
* lpa1*^*2*^	21.2 ± 0.8^#^	22.8 ± 0.5^#^	0.446 ± 0.004	0.394 ± 0.010
* lpa2*		20.1 ± 1.7		0.272 ± 0.004
* lpa3*		20.2 ± 0.4		0.311 ± 0.005
* lpa4*	13.8 ± 0.3	15.0 ± 0.1	0.263 ± 0.001	0.291 ± 0.003
*BAT 881 (C2)*	14.4 ± 0.5	17.2 ± 0.8	0.295 ± 0.002	0.260 ± 0.002
* 938*		17.0 ± 1.5		0.263 ± 0.009
* HP5/1*	19.1 ± 1.4	12.5 ± 0.2	0.516 ± 0.003	0.355 ± 0.003
* Meccano*	9.75 ± 0.3	12.6 ± 0.1	0.278 ± 0.001	0.216 ± 0.002
* Mercato*	9.69 ± 0.5	11.4 ± 0.1	0.332 ± 0.004	0.225 ± 0.001
* Pan 146*		18.2 ± 1.1		0.258 ± 0.010
* Pan 123*		15.9 ± 0.2		0.271 ± 0.004

*NM* not measured.

BAT 93 (C1) was used as control for *lpa1*. *lpa1*^*2*^*. lpa2*. *lpa3*. *lpa4*. MECCANO. MERCATO. PAN-123 and PAN-146. BAT-881(C2) as control for HP5/1 and 938. Significant differences against C1 are represented by (#) and for C2 (¤) Significant differences between 2021 and 2022 are represented by (*). Data = mean ± SEM; n = 3 for each group. For all symbols (*, #, ¤): *p < 0.05; **p < 0.01; ***p < 0.001; ****p < 0.0001.

### Correlation between ANF content and bioaccessibility/solubility of fat-soluble compounds and minerals.

**Figure 3:**
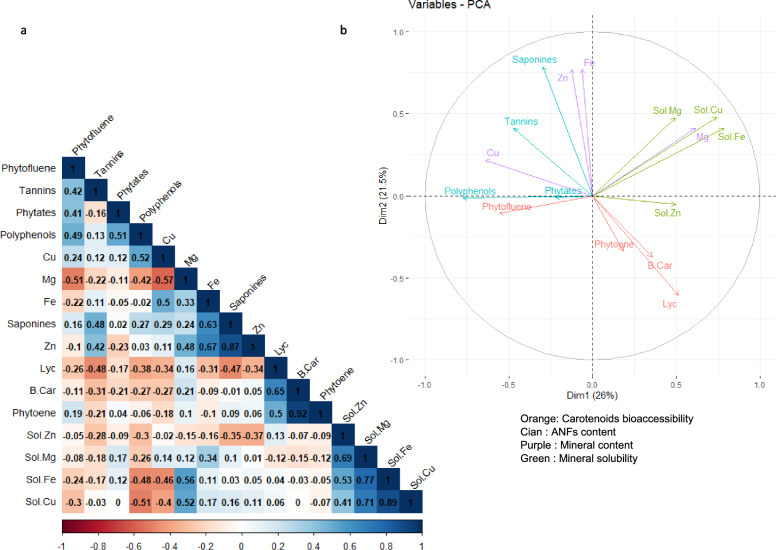
Relationship between carotenoid bioaccessiblity/ mineral solubility and bean content in minerals, phytates, saponins and tannins in 2021. A principal component analysis (PCA) was conducted on all samples to analyze the relationship between the presence of phytates, saponins, tannins and carotenoid bioaccessibility and mineral content/solubility (**b**). Correlation values are given in panel (**a**).

For 2021, the first and the second principal components of the PCA (Fig. 3a,b) explained 26% and 21.5% of the total variance, respectively. The first axis shows that total polyphenol contents are inversely correlated with the solubility of magnesium, iron and cupper. The second axis shows that tannin and saponin contents are inversely correlated with lycopene, phytoene and β-carotene bioaccessibility.

**Figure 4:**
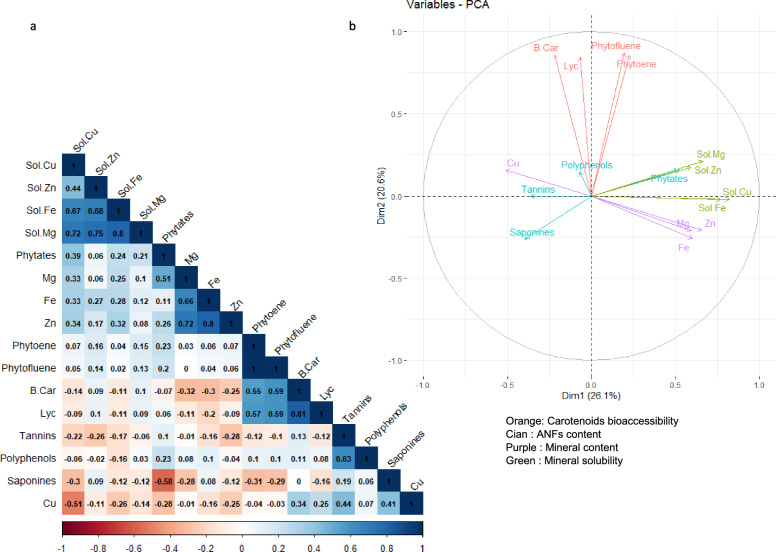
Relationship between carotenoid bioaccessiblity/ mineral solubility and bean content in minerals, phytates, saponins and tannins in 2022. A Principal Component Analysis (PCA) was conducted on all samples to analyze the relationship between the presence of phytates, saponins, tannins and carotenoid bioaccessibility and mineral content/solubility (**b**). Correlation values are given in panel (**a**).

For 2022 (Fig. 4a,b), the first and the second principal components of the PCA explained 26.1% and 20.6% of the total variance, respectively. The first axis also shows an inverse correlation between the solubility of minerals or mineral contents (except for copper) and the bean content in total polyphenols, tannins and saponins.

## Discussion

This study aimed at evaluating the impact of the ANF content of prepared beans on carotenoid bioaccessibility and mineral solubility.

The ANF contents given are those found in cooked beans. As soaking and cooking can reduce soluble ANF levels^10^, these contents are certainly lower than in raw beans.

This study was conducted with beans produced in 2021 and in 2022, as the nutritional and antinutritional composition of beans can vary from one year to another. For instance, Mercato beans contained less phytates and less saponins than BAT 93 in 2021, but not in 2022. These differences can partly be explained by the growth conditions and the stability of the bean profiles. The 2022 cropping season was characterized by high temperatures and low rainfall. Although plants were bred in experimental fields and subjected to controlled irrigation, dry years such as 2022 may influence the chemical composition of bean seeds. It is therefore not surprising to observe significant differences in the nutritional characteristics of seeds obtained in 2021 and 2022. However, despite these variations, the amount of ANFs determined generally confirmed the different nutritional profile of each bean variety. All *lpa* mutant beans had reduced phytate amounts. Some of these differences were not significant, but this might possibly be due to the number of samples analyzed.

The *lpa* mutant beans have been further developed for several reasons. The first reason was to improve mineral absorption. Indeed, phytic acid can bind to divalent cations like iron, zinc, and calcium, reducing their absorption in the digestive tract. The *lpa* beans may thus facilitate the absorption of these essential minerals. The negative effect of phytates has also been found for other micronutrients. We previously showed that phytates can decrease both vitamin D and K bioaccessibility and their uptake by enterocytes^8,9^. This can be particularly deleterious for the nutrition of populations in regions where beans are staple foods and where dietary deficiencies in minerals are common^32^.

Phaseolin and lectin variants were further studied because of their nutritional interest. Indeed, these variant beans had a lower content of the antinutrients lectins that can interfere with iron absorption^7^ and the absence of phaseolin can improve protein digestibility^33^. The HP5/1 line also had a reduced phytate content^33^.

Since bean ANFs cannot only impact on the bean’s micronutrient bioavailability but also on the bioavailability of micronutrient coming from the same meal, beans were also included in a micronutrient-rich recipe to study their effect on both mineral solubility and carotenoid bioaccessibility. Beans were associated to tomato and red bell pepper, two vegetables particularly rich in carotenoids^34,35^. Vitamin D-enriched oil was also added to the test meals. All test meals were digested using an in vitro digestion model previously validated to evaluate both fat-soluble compound bioaccessibility and mineral solubility^9^. Despite their low phytate content, *lpa* mutant beans did not improve the mineral solubility and carotenoid/vitamin D bioaccessibility. This result can have several explanations. The first one is that *lpa* mutant lines still contain phytates at a concentration that may be sufficient to have a negative effect on mineral solubility. The second reason might be that the *lpa* lines, as shown in our study, contain significant amounts of tannins, polyphenols and saponins. Both tannins, polyphenols and saponins have the ability to chelate or bind to minerals, forming complexes that are often insoluble, which consequently decrease mineral bioavailability^36^. Some tannins and polyphenols can also inhibit digestive enzymes, which can interfere with the proper digestion and absorption of micronutrients in the gastrointestinal tract^37,38^. Saponins further limit the incorporation of lipids into mixed micelles produced during digestion^39^ and haver a highly significant negative effect on vitamin K bioaccessibility, at least in vitro^8^. Finally, we did not quantify fibers in our study. These fibers are not ANFs, but fibers can impair the bioaccessibility of both minerals^8,10^ and fat-soluble micronutrients such as carotenoids^40^.

Our results differ from a previous clinical trial showing an increased iron bioavailability of common bean *lpa1* mutants in human volunteers^41^. In this study, the phytate content of the *lpa1* beans was 90% lower when compared to a control, while the polyphenol content remained similar. In our study, only *lpa1* showed a comparable decrease in phytates. However, even if *lpa1* mutant content in polyphenols was similar to the control bean line, *lpa1* saponin content was significantly higher than in the control bean line, which may impact on micronutrient bioaccessibility. Interestingly, an increased carotenoid bioaccessibility was obtained with HP5/1 beans when compared to their respective control BAT-881. This may be due to their low content in phytates in addition to low phaseolin and lectin. However, mineral solubility in the presence of HP5/1 beans was not improved compared to the control condition.

We then estimated zinc absorption by using a prediction equation^31^. Such prediction was not performed with iron as iron prediction equation requires to know the iron status of the individuals to be calculated^31^. When estimating zinc absorption from a portion of 200 g of *lpa* beans, it was predicted zinc increased twofold compared to its control. This increase that was not observed in vitro but we only estimated the solubility (i.e. the mineral found in the aqueous phase of the digesta after digestion) while the equation is supposed to predict the amount of zinc after digestion and absorption by the intestine. Such discrepancy can also derive from the fact that the zinc bioavailability equation does not consider polyphenols and tannins from the diet.

However, the PCA further confirmed a negative correlation between ANFs and both carotenoid bioaccessibility and mineral solubility, according to previous PCA analysis^8^.

Overall, our study indicates that the total ANF content of the mutated bean lines is still not sufficiently optimized. Further agronomical research is thus needed to improve the phytate content in bean seeds but also of other ANFs.

Additionally, evaluating the fiber content of beans, and to finely characterize in greater detail all polyphenols present in each bean line would be interesting and will be, therefore, a target for future studies.

Finally, we previously also showed that the ANF content of bean seeds can be decreased by optimizing their preparation process^10^. This decrease was, however, insufficient to improve fat-soluble micronutrient bioavailability. We, therefore, think that combining seed improvement with an optimized processing method can ultimately contribute to a significant improvement of micronutrient bioavailability from common beans.

## Data Availability

The data sets generated during and/or analysed during the current study are available from the corresponding author on reasonable request.
